# Clinical and Patient-Reported Outcomes Following Multidisciplinary Management of Cancer-Related Pain: Protocol for a Prospective Observational Pre-Post Study

**DOI:** 10.2196/92566

**Published:** 2026-08-27

**Authors:** Juan Jose Amate Pena, Laura García Reza, Patricia Concheiro-Moscoso, Betania Groba, Sara Sierra Cardalda, Clara García Lorenzo, Miguel Angel Pereira Loureiro

**Affiliations:** 1 Pain Unit Department of Anesthesiology and Pain Medicine University Hospital Complex Of Vigo Vigo, Pontevedra Spain; 2 Department of Anesthesiology and Pain Medicine University Hospital Complex Of Vigo Vigo, Pontevedra Spain; 3 Faculty of Health Sciences, Oza Campus Research Center for Information and Communication Technologies, Technology Applied to Research in Occupation, Equality and Health Group Universidade da Coruña A Coruña, Galicia Spain

**Keywords:** pain, cancer, multidisciplinary, patient, committees, clinical

## Abstract

**Background:**

Cancer-related pain remains highly prevalent and frequently undertreated despite advances in oncological therapies and analgesic strategies. Multidisciplinary approaches are recommended to optimize complex cancer pain management and integrate supportive care; however, robust real-world evidence evaluating their measurable clinical and patient-reported impact remains limited. Structured multidisciplinary cancer pain committees may be associated with changes in pain outcomes, quality of life, and patient experience, but prospective evaluations in routine clinical practice are scarce.

**Objective:**

The primary objective of this study is to describe changes in pain intensity following multidisciplinary evaluation in patients with cancer-related pain. Secondary objectives include describing changes in quality of life, pain interference, opioid consumption, treatment-related adverse effects, and patient-reported experience measures (PREMs) following multidisciplinary cancer pain committee assessment.

**Methods:**

This is a prospective, single-center, longitudinal, observational pre-post study conducted in a tertiary university hospital. A total of 68 adult patients with cancer-related pain referred to a multidisciplinary cancer pain management committee will be consecutively included in the study. Clinical variables and patient-reported outcomes will be collected at baseline (T0, before committee evaluation) and at 60 days following multidisciplinary committee assessment (T1). The primary outcome measure will be the change in pain intensity measured using the visual analog scale. Secondary outcomes include pain interference assessed with the Brief Pain Inventory–Short Form; health-related quality of life measured with the EQ-5D; and opioid consumption, treatment-related adverse effects, and PREMs, including patient satisfaction, assessed using the Likert scale. Pre-post comparisons will be performed using paired statistical tests (2-tailed paired *t* test or Wilcoxon signed rank test, depending on data distribution). Statistical significance will be set at *P*<.05.

**Results:**

Participant recruitment began in July 2026. At the time of submission of this revised manuscript, 3 participants had been enrolled. Recruitment and follow-up are ongoing. Data collection is expected to continue through late 2027, and the primary study results are expected to be available in early 2028, following completion of data collection and statistical analysis.

**Conclusions:**

This study will describe changes in clinical and patient-reported outcomes following multidisciplinary cancer pain committee assessment in routine clinical practice. Although causal inference is not possible because of the observational pre-post design, the findings may inform future optimization and evaluation of multidisciplinary cancer pain management pathways.

**Trial Registration:**

OSF Registries ty5m9; https://osf.io/ty5m9

**International Registered Report Identifier (IRRID):**

PRR1-10.2196/92566

## Introduction

### Background

Cancer-related pain is one of the most prevalent and debilitating symptoms in patients diagnosed with cancer and is likely the factor that most significantly affects and limits their quality of life [1]. The number of patients diagnosed with cancer continues to increase, as does cancer survival, largely owing to advances in diagnostic capabilities and oncological treatments; these improvements have contributed to increasing survival rates and, consequently, longer life expectancy after diagnosis [2]. As a consequence, this increased survival is associated with a higher burden of long-term adverse effects related both to the oncological disease itself and to its treatments, as well as with a growing number of patients experiencing cancer-related pain [3]. It is estimated that more than 50% of patients with cancer will experience pain at some point during the course of the disease [4], with this figure rising to 70% to 90% among patients with advanced disease, significantly affecting their physical, emotional, and social well-being.

According to the study by Sung et al [5], based on data analyzing 36 types of cancer across 185 countries, an estimated 19.3 million new cancer cases and 10 million cancer-related deaths occur worldwide each year. The most commonly diagnosed cancers were breast (11.7%), lung (11.4%), colorectal (10.0%), prostate (7.3%), and stomach cancer (5.6%). The cancers with the highest mortality were lung cancer (18.0% of all cancer deaths), colorectal cancer (9.4%), liver cancer (8.3%), stomach cancer (7.7%), and breast cancer (6.9%) [5]. Similar epidemiological trends have been reported in Spain according to data from the Spanish Network of Cancer Registries (REDECAN) [6]. With regard to metastases, although distribution depends on the type of primary tumor, the most common metastatic sites in descending order are lymph nodes, lungs, bones, and liver [7]. Bone is the third most common site of metastatic spread, particularly involving the axial skeleton (spine and pelvis) [8]. Bone metastases warrant special consideration due to their associated complications, especially pathological fractures and pain, which substantially compromise patients’ quality of life [9].

Cancer-related pain is usually somatic or mixed in nature and is less frequently purely neuropathic, although the type of pain depends on the characteristics of the oncological process. In addition to the tumor itself, oncological treatments such as chemotherapy, radiotherapy, and surgery may also be significant sources of pain. In the most recent revision of the World Health Organization’s (WHO’s) 11th revision of the International Classification of Diseases [10], cancer pain is defined either as chronic pain caused by the primary cancer or by metastases (chronic cancer pain), or as chronic pain resulting from cancer treatment, referred to as chronic postcancer treatment pain [11]. Several international organizations have developed clinical guidelines for the management of cancer-related pain, aiming to support optimal care, particularly in patients with advanced disease. In addition to the WHO analgesic framework, guidelines from the American Society of Clinical Oncology (ASCO) [12], the European Society for Medical Oncology (ESMO) [13], and the National Comprehensive Cancer Network (NCCN) [14] recommend individualized, multidisciplinary management, especially for patients with persistent, refractory, or complex pain requiring specialized interventions. These guidelines emphasize close collaboration among oncology, pain medicine, palliative care, rehabilitation, nursing, and psychosocial support services to optimize symptom control and deliver patient-centered care.

In general, most patients benefit from a multimodal approach that combines pharmacological treatment, interventional procedures, and physical and psychological therapies [3,15]. Pharmacological management has traditionally been based on the WHO analgesic ladder [16], which proposes a stepwise approach using nonopioid analgesics, weak opioids, and strong opioids, together with adjuvant medications according to the underlying pain mechanism (nociceptive, neuropathic, or mixed). However, this strategy has important limitations, particularly in patients with refractory pain, intolerance to opioid-related adverse effects, or localized pain that is difficult to control with systemic therapy [17]. Consequently, despite treatment with strong opioids and adjuvant medications, a substantial proportion of patients continue to experience uncontrolled pain or treatment-limiting toxicity [18]. In this setting, minimally invasive interventional procedures have become established as valuable adjuncts, especially in advanced or palliative disease. Available techniques, including nerve blocks, local anesthetic and corticosteroid injections, neuromodulation, and more recently ablative procedures, are selected according to the pain mechanism and underlying oncological condition to provide individualized pain relief whenever appropriate [19]. As cancer-related pain is frequently multifactorial and evolves throughout the disease trajectory, optimal management often requires coordinated multidisciplinary care. Accordingly, several models have been implemented in oncology practice, including specialized cancer pain clinics, palliative care consultation teams, integrated supportive care services, and multidisciplinary case conferences. Although their organizational structures differ, these models share the common goal of providing comprehensive assessment, facilitating shared clinical decision-making, optimizing pain management, and ensuring timely access to supportive and interventional therapies [20-24]. However, the scientific evidence supporting these organizational models remains limited. Most published studies have been retrospective observational studies or service evaluations performed in single institutions, with considerable heterogeneity in team composition, referral pathways, outcome measures, and follow-up. Consequently, the implementation and clinical outcomes associated with multidisciplinary cancer pain committees in routine clinical practice remain insufficiently characterized, highlighting the need for prospective observational studies evaluating patient outcomes after multidisciplinary assessment [25-28].

This study has therefore been designed as a prospective observational service evaluation protocol to characterize clinical outcomes, patient-reported outcomes, patient experience, and therapeutic decisions following multidisciplinary committee assessment. The multidisciplinary cancer pain management committee evaluated in this study is an established component of routine clinical care at our institution and was not created specifically for research purposes. The findings may enhance understanding of multidisciplinary cancer pain pathways and help inform future quality-improvement initiatives.

### Objectives

#### Primary Objective

The primary objective is to describe changes in pain intensity between baseline and 60 days following multidisciplinary committee assessment among patients with cancer-related pain managed in routine clinical practice.

#### Secondary Objectives

The secondary objectives are to assess patient-reported experience measures (PREMs) and patient-reported outcome measures (PROMs) [29] following multidisciplinary committee evaluation, to describe changes in opioid consumption measured as daily morphine equivalent dose (DMED), to characterize changes in treatment-related adverse effects associated with analgesic therapy, and to describe the therapeutic interventions proposed by the multidisciplinary committee.

Given the ethical and organizational nature of multidisciplinary cancer pain committees, randomization or withholding committee evaluation would not be feasible or ethically appropriate. A prospective pre-post design allows evaluation of clinically meaningful changes in pain and patient-reported outcomes while preserving routine clinical care. Although causal inferences cannot be made, this design is appropriate for evaluating the implementation of a multidisciplinary cancer pain management pathway in routine clinical practice.

## Methods

### Study Design and Setting

This is a prospective, observational, longitudinal pre-post study conducted at the Vigo University Hospital Complex, a tertiary care university hospital in Galicia, Spain.

### Study Timeline

Recruitment began in July 2026 and is expected to continue for approximately 18 months. At the time of submission of this revised manuscript, 3 participants had been enrolled. Final follow-up assessments are anticipated by late 2027, after which data analysis will be performed. Dissemination of the study findings through publication in a peer-reviewed journal is expected in early 2028.

### Study Population

The study comprises adult patients (aged ≥18 years) with histologically confirmed active cancer, defined as malignant disease requiring active antineoplastic treatment or with documented disease progression, who present with moderate to severe cancer-related pain (visual analog scale [VAS] ≥4) and are referred to the multidisciplinary cancer pain management committee. A VAS threshold of ≥4 was selected based on commonly accepted definitions of moderate pain intensity in oncology populations and its clinical relevance as an indication for treatment escalation [30].

### Study Procedures

#### Overview

The multidisciplinary cancer pain management committee constitutes part of routine clinical care and does not represent an experimental intervention. Participation in the study does not modify clinical decision-making or patient management. The study procedures comprise the following sequential steps as mentioned below.

#### Referral to the Multidisciplinary Committee

Patients are referred to the multidisciplinary cancer pain management committee by the treating physician whenever multidisciplinary assessment is considered clinically appropriate.

#### Multidisciplinary Committee Meeting

The committee meets monthly as part of routine institutional clinical practice. During each meeting, the referring physician presents the clinical case. Committee members review the patient’s oncological diagnosis, disease stage, ongoing cancer treatment, pain characteristics, previous analgesic management, imaging findings when available, and overall clinical status. Recommendations are established by multidisciplinary consensus after discussion of each case by all participating specialists. When different management options are considered appropriate, the final recommendation is reached through multidisciplinary agreement and documented in the patient’s clinical record.

#### Recruitment and Informed Consent

Patients meeting the eligibility criteria are invited to participate by the principal investigator during the first face-to-face visit to the pain unit following committee discussion. Written informed consent is obtained before study-specific data collection.

#### Baseline Assessment (T0)

Demographic characteristics, cancer-related variables, pain intensity, opioid consumption, quality of life, pain interference, adverse effects, committee recommendations, and their planned implementation are recorded.

#### Follow-Up

Patients continue receiving routine clinical care. Clinical follow-up is performed according to usual practice by the pain unit, either in person or by telephone depending on clinical needs. During follow-up, pain intensity, opioid treatment, adverse effects, implementation of committee recommendations, and clinically relevant events are documented.

#### Final Assessment (T1)

Sixty days after baseline, patients undergo the predefined outcome assessment, including pain intensity, quality of life, pain interference, opioid consumption, adverse effects, and PREMs.

The primary analysis will compare baseline (T0) and 60-day (T1) assessments (Table 1). Information collected during routine follow-up will primarily be used for clinical monitoring and descriptive analyses.

**Table 1:** Schedule of assessments and data collection time points.

Variable	Baseline (T0)	Routine clinical follow-up	60 days (T1)
Informed consent	✓		
Demographics	✓		
Cancer characteristics	✓		
VAS^a^	✓	✓	✓
EQ-5D	✓		✓
BPI-SF^b^	✓		✓
DMED^c^	✓	✓	✓
Adverse effects	✓	✓	✓
PREMs^d^ (satisfaction)			✓
Committee recommendations	✓		
Implementation of recommendations		✓	✓

^a^VAS: visual analog scale.

^b^BPI-SF: Brief Pain Inventory–Short Form.

^c^DMED: daily morphine equivalent dose.

^d^PREM: patient-reported experience measure.

### Study Outcomes

The primary outcome is the change in pain intensity, measured using the VAS, from baseline (T0) to 60 days (T1) following multidisciplinary committee assessment. A reduction of at least 1 point on the VAS was predefined as the minimal clinically important difference, based on previous literature in patients with cancer-related pain [31]. This threshold was used for the sample size calculation and will also serve to interpret the clinical relevance of the observed changes.

Secondary outcomes include changes between T0 and T1 in health-related quality of life measured using the EQ-5D [32], pain-related functional interference assessed with the Brief Pain Inventory–Short Form (BPI-SF) [33], DMED, analgesic treatment–related adverse effects, and PREMs, including satisfaction with care assessed using a Likert-scale questionnaire at T1.

Table 2 summarizes the study outcomes, instruments, and time points. The primary analysis will focus on paired comparison of VAS scores between T0 and T1. Secondary outcomes listed in Table 2 will be analyzed accordingly based on variable type and distribution.

**Table 2:** Study outcomes.

Outcome category	Variable	Instrument or source	Time points	Type
Pain intensity	VAS^a^ score (0-10)	VAS	T0 and T1	Primary
Quality of life	EQ-5D index score	EQ-5D questionnaire	T0 and T1	Secondary
Pain interference	BPI-SF^b^ interference score	BPI-SF	T0 and T1	Secondary
Opioid consumption	Daily morphine equivalent dose (mg/day)	Clinical record	T0 and T1	Secondary
Adverse effects	Presence or absence and type	Clinical assessment	T0 and T1	Secondary
Committee recommendation	Type of recommendation issued	Committee record	T0	Descriptive
Implementation of recommendations	Implementation status (implemented, partially implemented, or not implemented)	Electronic medical record	T1	Exploratory
Patient satisfaction	Likert-scale satisfaction score	PREM^c^ questionnaire	T1	Secondary

^a^VAS: visual analog scale.

^b^BPI-SF: Brief Pain Inventory–Short Form.

^c^PREM: patient-reported experience measure.

### Recruitment and Study Procedures

#### Patient Eligibility Criteria

##### Inclusion Criteria

Inclusion criteria include adults aged ≥18 years with active cancer, defined as histologically confirmed malignant disease receiving active antineoplastic treatment or with documented disease progression; moderate to severe cancer-related pain (VAS ≥4); presentation to the multidisciplinary cancer pain management committee; ability to understand study procedures in Spanish or Galician; and provision of written informed consent.

##### Exclusion Criteria

Exclusion criteria include patients with severe cognitive impairment, dementia, intellectual disability, or any other condition preventing reliable completion of study assessments; patients in the agonal phase or with an expected survival insufficient to complete follow-up; refusal or inability to provide written informed consent.

#### Recruitment

**Figure 1:**
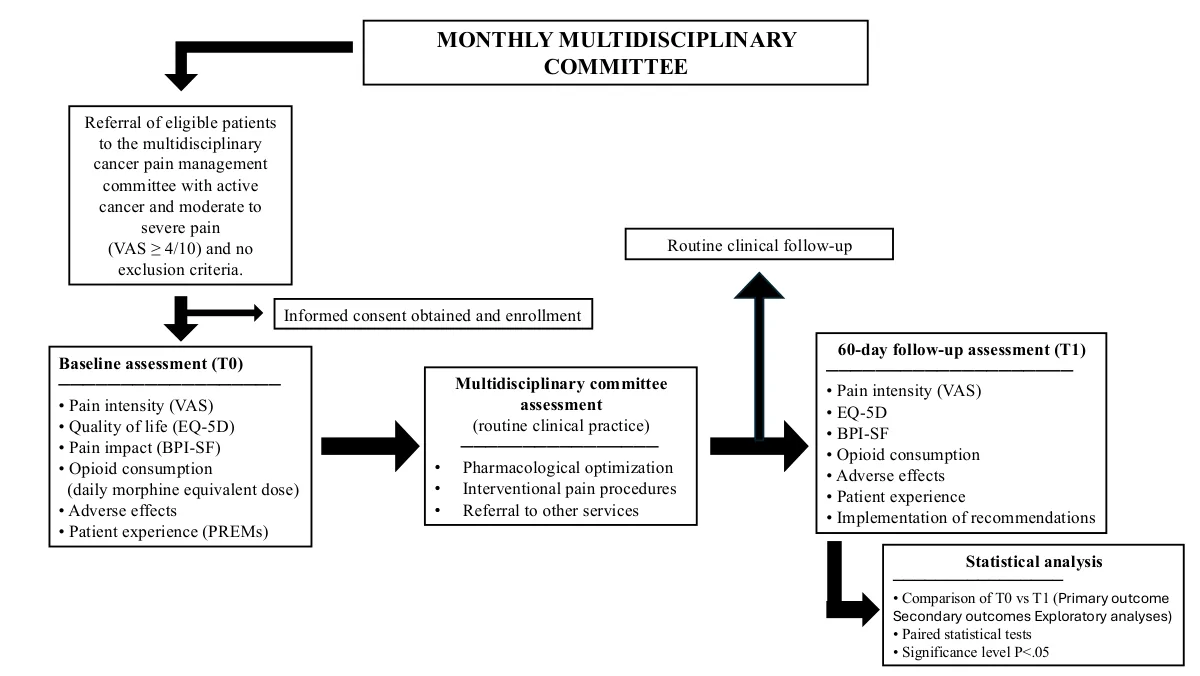
Planned participant flow for the prospective observational pre-post study. BPI-SF: Brief Pain Inventory–Short Form; PREM: patient-reported experience measure; VAS: visual analog scale.

All eligible patients meeting the inclusion criteria and none of the exclusion criteria will be consecutively invited to participate in the study. Referral to the multidisciplinary cancer pain management committee may be initiated by any physician involved in the care of adult patients with cancer. Referrals are most commonly made by specialists in medical oncology, radiation oncology, pain medicine, hematology, palliative care, or other hospital-based specialties managing patients with cancer. Referral is made at the treating physician’s discretion whenever multidisciplinary assessment is considered clinically appropriate. The multidisciplinary committee meets once monthly, and each meeting lasts approximately 1 hour. Clinical cases are presented by the referring physician and discussed by a multidisciplinary panel, including specialists in pain medicine, medical oncology, radiation oncology, and pain nursing. Additional specialists may participate whenever considered clinically appropriate. Management recommendations are established by multidisciplinary consensus after review of the patient’s oncological history, pain characteristics, current oncological treatment, previous analgesic strategies, imaging findings when available, and overall clinical condition. Recommendations may include optimization of pharmacological therapy, opioid rotation or dose adjustment, interventional pain procedures, referral for radiotherapy, referral to other medical specialties, psycho-oncology assessment, rehabilitation, palliative care interventions, or additional diagnostic evaluation according to individual patient needs. Implementation of committee recommendations will be assessed by reviewing the patient’s electronic medical record during follow-up and categorized as fully implemented, partially implemented, or not implemented. Written informed consent will be obtained by the principal investigator during the first face-to-face visit to the pain unit after multidisciplinary committee evaluation. For hospitalized patients, informed consent will be obtained during a bedside visit following committee discussion. Patients will be informed that participation is entirely voluntary and that declining participation or withdrawing from the study will not affect their routine clinical care or any therapeutic decisions made by the multidisciplinary committee. Baseline assessments (T0) will be completed as close as possible to multidisciplinary committee assessment, according to routine clinical practice. Participants will subsequently be followed for 60 days, with the primary end point assessed at T1. Figure 1 summarizes the participant flow throughout the study.

#### Data Management and Security

Clinical data will be collected using a study-specific electronic case report form. All data will be coded and pseudonymized to protect participant identity. Identifiable personal data will be stored separately from clinical data and secured in locked facilities within the responsible health care institution, with access restricted to authorized members of the research team. Only the research team and competent health authorities, who are bound by confidentiality obligations, will have access to the study data. Data processing, storage, communication, and transfer will be carried out in accordance with the General Data Protection Regulation (Regulation (EU) 2016/679) and relevant national data protection laws. The health care institution where data are collected will act as the data controller. Upon completion of the study, data will be anonymized for future research use when participants have provided explicit consent; otherwise, the data will be securely destroyed.

### Data Monitoring

Given the observational nature of the study and the absence of experimental interventions, no independent data monitoring committee was established. Data quality will be reviewed monthly by the principal investigator according to a predefined quality control checklist to verify completeness, internal consistency, and accuracy of data entry. Any discrepancies identified during quality control procedures will be resolved through review of the original clinical records before database locking.

### Statistics

#### Sample Size and Power Calculations

The sample size calculation was based on the primary outcome, defined as the change in VAS score between baseline and 60 days. The calculation was based on a paired comparison of means. A reduction of at least 1 point on the VAS was considered clinically relevant. Assuming a significance level of 5% (α=.05), a statistical power of 80% (β=.20), and an SD of 2, a total of 64 patients would be required to detect statistically significant differences. Allowing for an anticipated 5% dropout rate, a total sample size of 68 patients will be recruited.

#### Statistical Analysis Plan

Statistical analyses will be performed using SPSS (IBM Corp) or equivalent statistical software. Baseline demographic and clinical characteristics will be summarized using appropriate descriptive statistics. Variables of interest will include age, sex, cancer type, tumor stage, presence of metastatic disease, ongoing oncological treatment, baseline opioid use (DMED), previous interventional pain procedures, and palliative care status. A 2-sided *P* value of <.05 will be considered statistically significant. Given the exploratory nature of secondary outcomes, no formal adjustment for multiple comparisons will be applied; therefore, secondary analyses will be interpreted cautiously.

Categorical variables will be summarized as frequencies and percentages. Continuous variables will be described using means and SDs or medians and IQRs, depending on data distribution.

Normality of continuous variables will be assessed using the Shapiro-Wilk test to determine the appropriate statistical test.

For the primary outcome, VAS scores at baseline (T0) and at 60 days (T1) will be compared using paired *t* tests or Wilcoxon signed rank tests, as appropriate. Mean changes in VAS scores will be reported together with 95% CIs and standardized effect sizes (Cohen *d*) to facilitate interpretation of both statistical and clinical significance.

Secondary pre-post comparisons for continuous variables (eg, EQ-5D scores, BPI-SF scores, and DMED) will be analyzed using the same approach. Categorical variables, including adverse effects and selected PREMs, will be analyzed using chi-square or McNemar tests, as appropriate. Because pain outcomes may be influenced by patient and disease characteristics, exploratory multivariable regression analyses will be performed to evaluate the association between multidisciplinary committee assessment and changes in pain intensity after adjustment for clinically relevant covariates. Prespecified candidate covariates will include age, sex, cancer type, tumor stage, metastatic disease, baseline VAS score, baseline opioid consumption, ongoing oncological treatment, interventional pain procedures, and palliative care status.

Because of the observational design, the relatively small sample size, and the exploratory nature of these analyses, adjusted regression models will be considered hypothesis-generating and interpreted with appropriate caution. Predefined exploratory subgroup analyses will be performed whenever sample size permits. These analyses may include comparisons according to cancer type, metastatic vs nonmetastatic disease, baseline pain severity (moderate vs severe), and patients who are receiving palliative vs nonpalliative care. These subgroup analyses will be considered exploratory and interpreted cautiously.

Analyses will primarily be conducted using complete-case analysis. The frequency, pattern, and potential reasons for missing data will be described. In patients with advanced cancer, missing outcome data may result from clinical deterioration, hospitalization, or death and may therefore be informative (missing not at random). If missing data for the primary outcome exceed 5%, multiple imputation will be considered only as part of sensitivity analyses and only if the missing-data mechanism is considered reasonably compatible with the missing-at-random assumption. The results of any imputation-based analyses will be interpreted cautiously in light of the potential for informative missingness. Additional sensitivity analyses, including complete-case analyses, will be performed whenever appropriate to assess the robustness of the primary findings. All analyses will follow the predefined statistical plan approved by the research ethics committee.

As this is an observational study protocol rather than a clinical trial, the manuscript was primarily prepared in accordance with the STROBE (Strengthening the Reporting of Observational Studies in Epidemiology) statement [34]. Elements of the SPIRIT (Standard Protocol Items: Recommendations for Interventional Trials) statement were considered where applicable to improve protocol transparency [35].

A participant flow diagram will be used to describe progression through the study, including numbers assessed for eligibility, enrolled, completing follow-up, and included in the final analysis.

### Ethical Considerations

The study was reviewed and approved by the Comité de Ética de la Investigación de Pontevedra-Vigo-Ourense (Reference No. 2025/517). Ethics approval was granted in January 2026. Participant recruitment began in July 2026. The study will be conducted in accordance with the ethical principles of the Declaration of Helsinki (1964) and its subsequent revisions, including the 2024 revision, as well as the Convention on Human Rights and Biomedicine (Oviedo Convention, 1997). The study will also comply with applicable European and national regulations governing biomedical research. All procedures performed in this study are part of routine clinical practice, and no additional risks are anticipated for participants because of their involvement. Written informed consent will be obtained from all participants prior to inclusion in the study.

### Study Registration

The study protocol and statistical analysis plan were prospectively preregistered at the Open Science Framework (OSF) prior to participant recruitment (registration date: February 27, 2026). The preregistration is available on OSF.

## Results

Participant recruitment began in July 2026. At the time of submission of this revised manuscript, 3 participants had been enrolled. Recruitment and follow-up are ongoing. Data collection is expected to continue through late 2027, after which data analysis will be performed. The primary study results are expected to be available in early 2028, following completion of data collection and statistical analysis. Study findings will be disseminated through publication in a peer-reviewed journal and presentation at scientific conferences.

## Discussion

### Principal Findings

This protocol describes a prospective observational service evaluation study designed to characterize changes following assessment by a multidisciplinary cancer pain committee in routine clinical practice. Rather than determining treatment effectiveness, the study aims to describe changes in pain, quality of life, opioid use, adverse effects, patient-reported outcomes, and multidisciplinary management decisions following routine committee evaluation.

Through prospective collection of standardized information on pain intensity, quality of life, pain interference, opioid consumption, treatment-related adverse effects, and patient experience, this study seeks to provide a comprehensive description of patient outcomes following multidisciplinary management. Because of the observational pre-post design, causal relationships cannot be inferred, and the study is intended to characterize changes observed in a real-world health care setting.

### Comparison With Previous Literature

Current international recommendations from ASCO, ESMO, and NCCN advocate multidisciplinary management for patients with complex cancer-related pain; however, prospective evaluations of structured multidisciplinary cancer pain committees remain scarce. Most available studies have been retrospective, descriptive, or focused on specific therapeutic interventions rather than on the overall multidisciplinary decision-making process. By prospectively evaluating both clinical outcomes and patient-reported outcome and experience measures, this study may contribute additional evidence regarding multidisciplinary cancer pain management pathways in routine clinical practice.

### Strengths and Limitations

Strengths of this protocol include its prospective observational design, prospective protocol registration, consecutive patient recruitment, standardized outcome assessment, and evaluation of both clinical and patient-reported outcomes under routine clinical conditions. Furthermore, the multidisciplinary nature of the committee reflects contemporary recommendations for the management of complex cancer-related pain.

Several limitations should also be acknowledged. Because of the observational pre-post design and the absence of a control group, causal inference regarding the effect of multidisciplinary committee assessment cannot be established. Changes in pain intensity may also be influenced by regression to the mean, the natural course of the underlying disease, disease progression or response to oncological treatment, medication adjustments, or other concurrent clinical interventions occurring during follow-up. In addition, the single-center design may limit external validity, and residual confounding cannot be completely excluded despite planned exploratory adjusted analyses.

### Future Directions

The findings of this study may inform the design of larger multicenter prospective studies evaluating multidisciplinary cancer pain management pathways. Future investigations incorporating comparator groups and longer follow-up periods may further clarify factors associated with changes in patient outcomes and facilitate optimization of multidisciplinary models of care.

### Dissemination Plan

This study will provide evidence that will be disseminated through presentations at scientific conferences as well as publications in specialized national and international peer-reviewed journals, regardless of study outcomes. Furthermore, the findings may improve understanding of the role of multidisciplinary teams in the management of cancer-related pain and may help inform future clinical protocols and practice guidelines.

### Conclusions

This protocol describes a prospective observational evaluation of multidisciplinary cancer pain management implemented in routine clinical practice. The study is expected to characterize patient-reported outcomes, clinical outcomes, and multidisciplinary decision-making following committee assessment. Although causal inference is not possible with this design, the findings may support future optimization and further evaluation of multidisciplinary cancer pain management pathways.

## Data Availability

Deidentified participant data will be available from the corresponding author upon reasonable request and subject to institutional data protection regulations.

## Abbreviations

ASCO: American Society of Clinical Oncology
BPI-SF: Brief Pain Inventory–Short Form
DMED: daily morphine equivalent dose
ESMO: European Society for Medical Oncology
NCCN: National Comprehensive Cancer Network
OSF: Open Science Framework
PREM: patient-reported experience measure
PROM: patient-reported outcome measure
REDECAN: Spanish Network of Cancer Registries
SPIRIT: Standard Protocol Items: Recommendations for Interventional Trials
STROBE: Strengthening the Reporting of Observational Studies in Epidemiology
VAS: visual analog scale
WHO: World Health Organization
